# Cold Adaptation, Ca^2+^ Dependency and Autolytic Stability Are Related Features in a Highly Active Cold-Adapted Trypsin Resistant to Autoproteolysis Engineered for Biotechnological Applications

**DOI:** 10.1371/journal.pone.0072355

**Published:** 2013-08-12

**Authors:** Alvaro Olivera-Nappa, Fernando Reyes, Barbara A. Andrews, Juan A. Asenjo

**Affiliations:** Centre for Biochemical Engineering and Biotechnology, Department of Chemical Engineering and Biotechnology, University of Chile, Santiago, Chile; National Institute for Medical Research, Medical Research Council, London, United Kingdom

## Abstract

Pig trypsin is routinely used as a biotechnological tool, due to its high specificity and ability to be stored as an inactive stable zymogen. However, it is not an optimum enzyme for conditions found in wound debriding for medical uses and trypsinization processes for protein analysis and animal cell culturing, where low Ca^2+^ dependency, high activity in mild conditions and easy inactivation are crucial. We isolated and thermodynamically characterized a highly active cold-adapted trypsin for medical and laboratory use that is four times more active than pig trypsin at 10^°^ C and at least 50% more active than pig trypsin up to 50^°^ C. Contrary to pig trypsin, this enzyme has a broad optimum pH between 7 and 10 and is very insensitive to Ca^2+^ concentration. The enzyme is only distantly related to previously described cryophilic trypsins. We built and studied molecular structure models of this trypsin and performed molecular dynamic calculations. Key residues and structures associated with calcium dependency and cryophilicity were identified. Experiments indicated that the protein is unstable and susceptible to autoproteolysis. Correlating experimental results and structural predictions, we designed mutations to improve the resistance to autoproteolysis and conserve activity for longer periods after activation. One single mutation provided around 25 times more proteolytic stability. Due to its cryophilic nature, this trypsin is easily inactivated by mild denaturation conditions, which is ideal for controlled proteolysis processes without requiring inhibitors or dilution. We clearly show that cold adaptation, Ca^2+^ dependency and autolytic stability in trypsins are related phenomena that are linked to shared structural features and evolve in a concerted fashion. Hence, both structurally and evolutionarily they cannot be interpreted and studied separately as previously done.

## Introduction

Trypsins (EC 3.4.21.4) are fairly specific endoproteases that hydrolyze peptide bonds after arginine or lysine residues, with less activity when either is followed by a proline residue [1]. Trypsins are readily available and commonly used in biological research during proteomics experiments where their high specificity is well suited for controlled and predictable proteolysis. They are also used in the processing of recombinant proteins [2–4] and as wound debridement agents and epidermal ablation treatments, alone or in combination with other proteases or chemicals [5–10]. One of the most important uses of trypsins is to re-suspend cells adherent to the cell culture dish wall during the process of harvesting cells in animal cell cultures [11]. Trypsin is used with EDTA to cleave Ca^2+^- and Mg^2+^-dependent integrins bonding the cultured cells to the dish, so that the cells can be suspended in fresh solution and transferred to fresh dishes [11–14]. Since trypsin activity and stability is Ca^2+^ dependent, the use of EDTA makes it necessary to increase the amount of enzyme and this uncontrolled treatment could alter the physiology, protein expression and metabolism of cultured cells [15–17].

Pig trypsin (PT) is the main enzyme used for these applications. These are usually carried out at temperatures lower than the PT optimal temperature. Temperatures lower than 15^°^ C in the processing of recombinant proteins, especially therapeutic proteins, are desirable to avoid proliferation of contaminating microorganisms. Many of these processes have low or no Ca^2+^ in the reaction medium. Fine-tuning and control of the enzymatic activity is also required to guarantee the correct amount of proteolysis and avoid unspecific hydrolysis. Hence, pig trypsin (PT) is clearly not optimal for these applications.

The specific activity of cold-active or psychrophilic enzymes is higher than that of their mesophilic counterparts between 0 and 30°C [18–21], which can be exploited for industrial and commercial purposes [22,23]. Enzymes adapted to low temperatures (< 10° C) have a lower activation energy and a high catalytic efficiency (k_cat_/K_M_) [19,24–26]. They are rapidly inactivated at medium-high temperatures, a phenomenon known as thermal instability. This can be advantageously used to tightly control their proteolytic activity in biotechnological applications.

Serine and trypsin-like proteases from Antarctic krill (*Euphausia superba*), a psychrophilic crustacean, have been previously purified and characterized [27–30]. We describe the cloning and engineering of a krill trypsin able to overcome the practical limitations of PT. Mutant proteins were designed and tested to improve the autolytic stability of the enzyme. The best mutant combines a long lasting high proteolytic activity with the thermal instability of a cold-adapted enzyme to tightly control the enzymatic activity.

## Materials and Methods

### Ethics statement

The Chilean Antarctic Institute (INACh) issued the permission to collect Antarctic krill (*Euphausia superba*) samples from the coast adjacent to the Frei Montalva Base (Lat 62° 11” S Long 58° 58″ W) at King, George Island, Chilean Antarctic Territory, on January 2003. Field studies did not involve endangered or protected species.

### Reagents

Restriction enzymes were supplied by New England Biolabs (US). Primers were supplied by Integrated DNA Technologies, Inc. (US), Taq DNA polymerase by Promega (US), Elongase and T4 DNA ligase by Invitrogen (US). Other reagents, culture media additives and solvents were of analytical grade.

### Strains, vectors, and culture medium

Antarctic krill (*Euphausia superba*) was collected in summer and stored in liquid nitrogen.

pGEM-T Easy (Promega) was used for subcloning in *E. coli* DH5α (Invitrogen). *E. coli* TB1 (New England Biolabs) and BL21(DE3) (Novagen Inc.) were used as bacterial hosts. pMAL-c2E (New England Biolabs) and *E. coli* BL21(DE3) (Novagen Inc.) were used expression vectors.

### Protein isolation, purification and partial amino acid sequencing

Sample preparation and purification was done according to Salamanca et al. [30]. Peptides from a purified fraction IV protease were sequenced. Four degenerate and two specific primers were designed based on the conserved peptide sequences obtained from similarity searches and used for RT-PCR amplifications.

### RNA extraction, cDNA cloning, and sequencing of the protease gene

Total RNA was isolated from frozen Antarctic krill with Trizol reagent (Invitrogen). The Oligotex.RTM kit (QIAGEN, US) was used to purify mRNA prior to cDNA synthesis by RT-PCR with Superscript II reverse transcriptase, and subcloning. A 5´ RACE and a 3´ RACE kit (Invitrogen) were used for amplification of the complete trypsin genes.

### Bioinformatic methods

Similarity searches were performed using pBLAST [31]. Multiple protein alignments were built using Clustal Omega [32]. Phylogenetic trees were constructed and visualized with TreeView 1.6. Protein structural models were built using the Robetta Server [33–36] and Modeller [37,38] and visualized with PyMol (www.pymol.org). Structural models of mutants were built using Andante [39]. The selection of non-disruptive mutations was performed using MOSST [40]. Prediction of the effect of mutations was carried out by MOSST and SDM [41]. Energy calculations were performed using the Gromos96 [42] implementation of Swiss-PDBViewer [43].

### Expression of a krill trypsin gene in *E. coli* cells

pMAL-c2E and pET-22b(+) were used as expression vectors for KT1 in *E. coli*. The two recombinant plasmids (pET22b-KT1 and pMALc2E-KT1) were electroporated into *E. coli* TB1 or BL21(DE3) cells using a Cell-Porator® System (GIBCO-BRL). Plasmids in transformants were characterized by restriction mapping and sequencing.

### Purification of an active recombinant krill trypsin

Recombinant *E. coli* strains were grown overnight at 37° C. After re-inoculating in fresh medium and adding IPTG, the temperature was lowered to 20° C and cells were harvested and processed after 2 hours. Soluble proteins were recovered from pMAL construct cultures by sonication and centrifugation, loaded onto an amylose resin column (New England Biolabs), washing and eluting the fusion maltose-binding protein (MBP)-trypsin with maltose. Inclusion bodies were recovered from the pET22b construct cultures by sonication and centrifugation. Purification and renaturation were performed following the QIAexpressionist System (QIAGEN) instructions. The zymogen produced by pET22b constructs was activated by digestion with a catalytic amount of pig trypsin. In pMALc2E constructs, zymogens were activated by digestion with either enterokinase or pig trypsin, which also removed the maltose-binding domain (MBD).

### Protein characterization

Protein fractions were analyzed by 12% SDS-PAGE and stained with CBB. Protein concentration was determined according to Bradford [44] with ovalbumin as the standard. Unspecific protease activity was determined by incubating a protein preparation with largely saturating concentrations of casein (or hemoglobin at pH<6.0). The release of fragments was measured using the Folin-Ciocalteu method. Unspecific protease activities were compared to succinylated casein standards to express the result in suitable units. Trypsin-like activity was determined by measuring the degradation of Nα-Benzoyl-L-arginine 4-nitroanilide (BAPNA).

Active trypsin bands were identified by zymographic analysis on a non-denaturating 12% PAGE with 1% gelatin in the gel. Samples were applied without previous heating in a load buffer with SDS and without β-mercaptoethanol. After electrophoresis, the enzyme was renatured and gels were stained with CBB.

### Western blot analysis

The SuperSignal® West HisProbe™ Kit (Pierce Biotechnology) was used to identify polyhistidine tagged protein bands (15-50 μg of protein per sample). Bands were detected by chemoluminescence.

## Results and Discussion

### Purification and characterization of the cold active krill proteases

**Figure 1:**
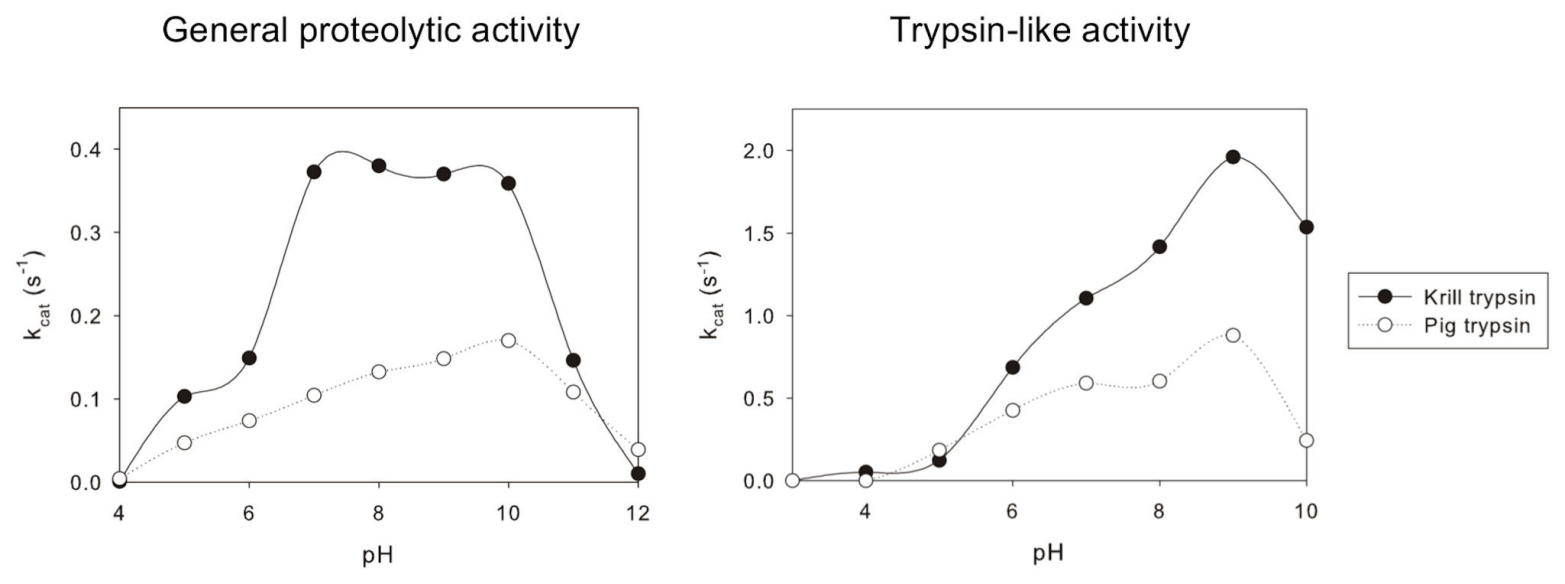
pH dependency of the catalytic activity of the purified krill trypsin. Comparative plots of enzymatic activity versus pH for the isolated cryophilic krill trypsin and the mesophilic pig trypsin.

Following our previously published protocols [30], we isolated several trypsin-like protein fractions from a krill autolysate, using BAPNA as a trypsin-specific substrate. We purified an enzyme with the highest activity and broadest pH-activity profile, useful features for practical applications. The pH optimum of the isolated protein was substrate dependent. For casein/hemoglobin, the pH optimum showed a plateau between 7 and 10, while for BAPNA, the pH optimum showed a distinct maximum around 9 (Figure 1).

**Figure 2:**
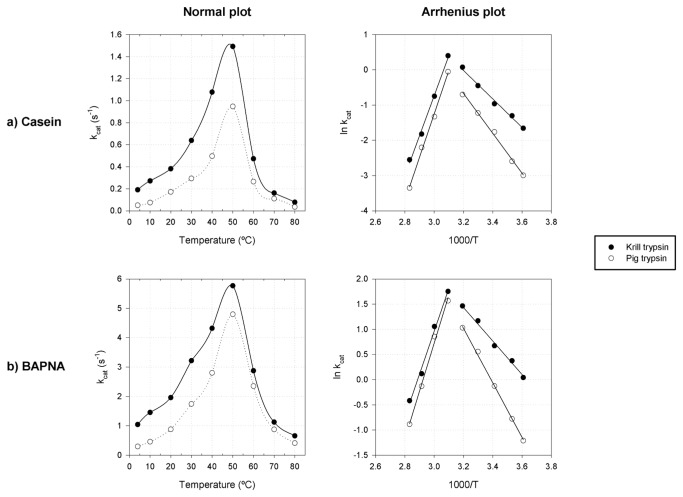
Temperature dependency of the catalytic activity of the purified krill trypsin. Comparative plots of enzymatic activity versus temperature in the range from 4 to 80^°^C for the isolated cryophilic krill trypsin and the mesophilic pig trypsin on two substrates, casein (A) and BAPNA (B). The corresponding Arrhenius plots are also shown, including the corresponding linear regressions at each side of the maximum.

The purified krill trypsin (KT) consistently showed higher catalytic constants than PT (used as a control) at its optimum pH in all the temperature range (4-80^°^C), reaching more than 400% of PT activity at ≤10^°^ C (Figure 2). KT showed a maximal trypsin-like activity k_cat_ of ~6 s^-1^ (^≈^ 16 U/mg) and a maximal caseinolytic activity k_cat_ of ~1.5 s^-1^ (^≈^ 40 U/mg), both around 50^°^ C, as shown in Figure 2.

KT and PT activation energies for both substrates were calculated from the Arrhenius plots (Figure 2; Table S1). The KT caseinolytic activation energy is ~70% of the activation energy of the mesophilic PT. For trypsin-like activities, KT showed an ~40% lower activation energy than PT. Despite these differences, free energies of activation (ΔG^#^) are very similar for both enzymes and substrates at 20^°^ C (~70-75 kJ/mol). Activation enthalpies are lower in KT for both substrates (Δ(ΔH^#^)_p-m_ < 0), but this gain in catalytic activity is counteracted by a similar increase in the entropic term (TΔ(ΔS^#^)_p-m_ < 0). The negative value of the entropic contribution has been associated with an increased flexibility of cryophilic enzyme structures and, together with lower enthalpic terms, are intrinsically associated with the cold adaptation of enzymes [45]. The net result is a Δ(ΔG^#^)_p-m_ of minus 1.3-1.4 kJ/mol between the psychrophilic and the mesophilic enzyme reaction for both substrates, which is the main factor behind the higher k_cat_ values of KT and its higher activities at low temperatures. All the above thermodynamic characterization demonstrates the cryophilic nature of KT [19,20,45,46].

### Isolation, cloning and sequencing of the KT gene

The purified KT was digested, peptides sequenced and five highly conserved amino acid regions were identified in homologous Crustacean trypsins. Primers were designed to amplify the KT gene from krill cDNA, giving a first 532-bp amplicon corresponding to a 177 amino acid peptide. RACE amplifications and sequencing yielded 3’ and 5’ fragments that were used to reconstruct the complete KT gene sequence *in silico*. A 236-amino acid ORF was identified in the reconstructed sequence, sharing a 78% identity with the closest *L* *. vannamei* trypsin [47] at the amino acid level. Amplification of the complete KT cDNA sequence with new primers provided two different 795 bp isoform sequences, named KT1 and KT4 (GenBank accession numbers ABS93146.1 and ABS93147.1; US Patent 7,202,074).

KT1 and KT4 have 98% identity at the amino acid sequence level. The deduced protein sequences comprise 266 amino acids and include a 29-aa predicted pre-pro-precursor peptide with only two amino acid substitutions (K25R, D26G) between both isoforms. SignalP 3.0 indicated a 100% probability that an N-terminal 15-aa eukaryotic extracellular secretion signal peptide (MKGFVICLLVAGACA) is present.

The next 14 N-terminal amino acids are a trypsin activation domain that must be cleaved to produce a mature active enzyme having the conserved IVGG N-terminal sequence. In vertebrate trypsins, the activation domain is shorter (6 to 8-aa), but the 14-aa KT activation domain is conserved in several Arthropoda and Crustacean species [47–50].

Active KT1 and KT4 comprise 236 amino acid residues with a molecular mass of 25,056 Da and 25,071 Da respectively, closely matching those obtained by MALDI-MS by Sjődahl et al. [29]. Our purified KT fraction showed a molecular mass in SDS-PAGE around 30 kDa, which is consistent with previous findings [30,51]. The calculated theoretical isoelectric points [52] of KT1 and KT4 were around 3.5, very close to previous experimentally determined values [30].

### Structural model of the KT1 gene product

#### Phylogenetic analysis

**Figure 3:**
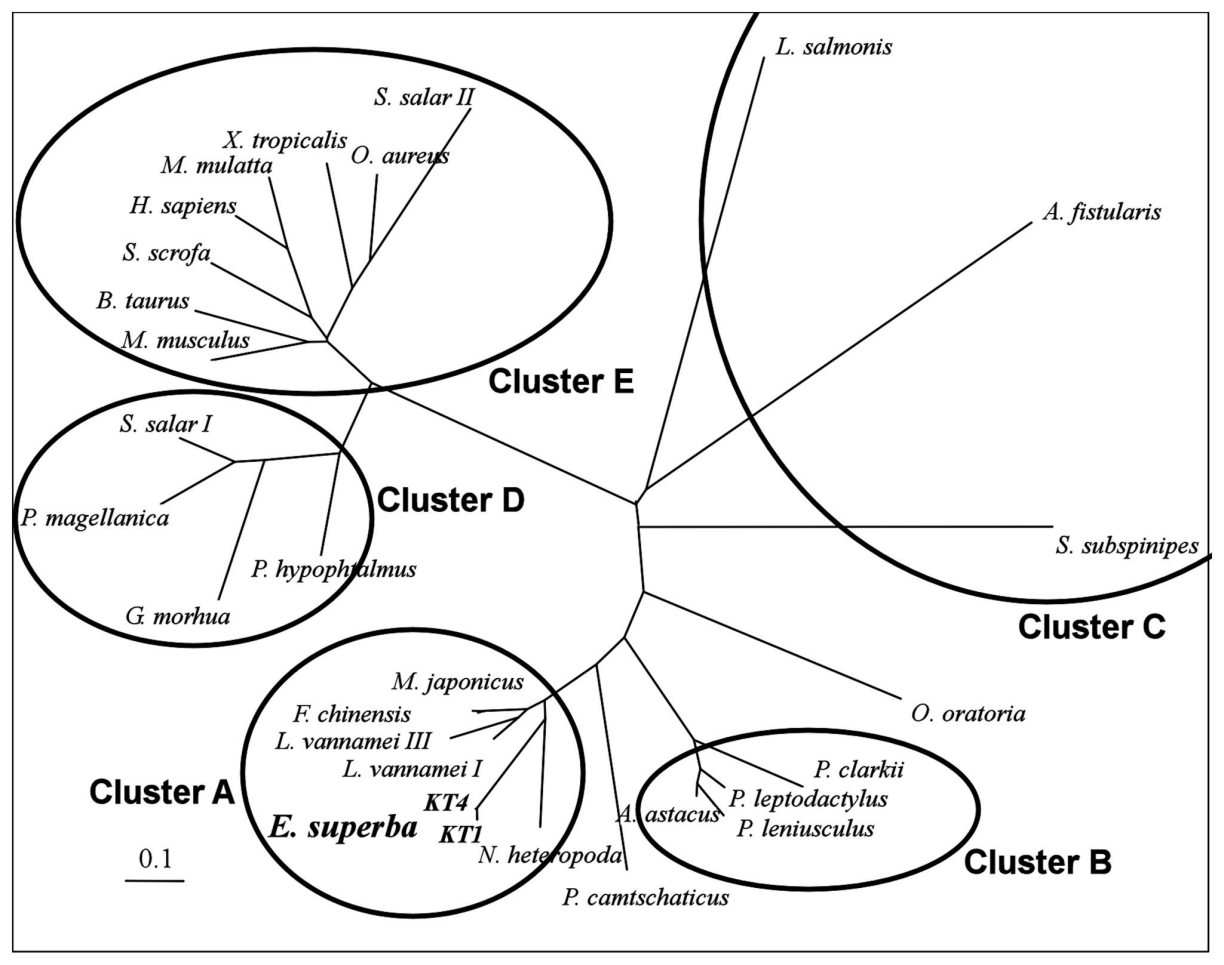
Unrooted phylogenetic tree of 28 trypsins inferred from their amino acid sequence alignment. Five phylogenetically-related clusters were inferred from this analysis: cluster A of shrimp and prawn trypsins (both cryophilic and mesophilic), cluster B of crayfish trypsins, cluster C of other non-crustacean Arthropoda, cluster D of cryophilic vertebrate trypsins, and cluster E of trypsins from mesophilic vertebrates. Cluster A trypsin sequences are shown to be very closely related to krill trypsins KT1 and KT4.

A phylogenetic tree (Figure 3) was constructed from mesophilic and psychrophilic trypsins from vertebrates and invertebrates with at least 30% identity with the krill sequences. Crustacean trypsins are evolutionarily separated from both mesophilic and psychrophilic vertebrates, and from other non-crustacean invertebrates*.*

Trypsins from shrimps and prawns (cluster A) show a high degree of sequence identity, independently of the temperature of their habitat. The most identical sequences to krill trypsins are those of *L* *. vannamei* , a mesophile that inhabits tropical waters at more than 20^°^ C all year round [53] and hence should not possess cryophilic enzymes. Krill trypsins do not have an appreciable degree of similarity with cold-adapted trypsins from marine fish. They are more similar to trypsins from Crustaceans (63-68% identity) and non-Crustacean Arthropoda (50% identity), and to a lesser extent with mammalian trypsins. This indicates that cold adaptation in this protein family is related to a small number of changes and not to changes in large molecular regions, and has taken a different evolutionary route from that of fish psychrophilic trypsins.

#### General features of the molecular model of KT1

**Figure 4:**
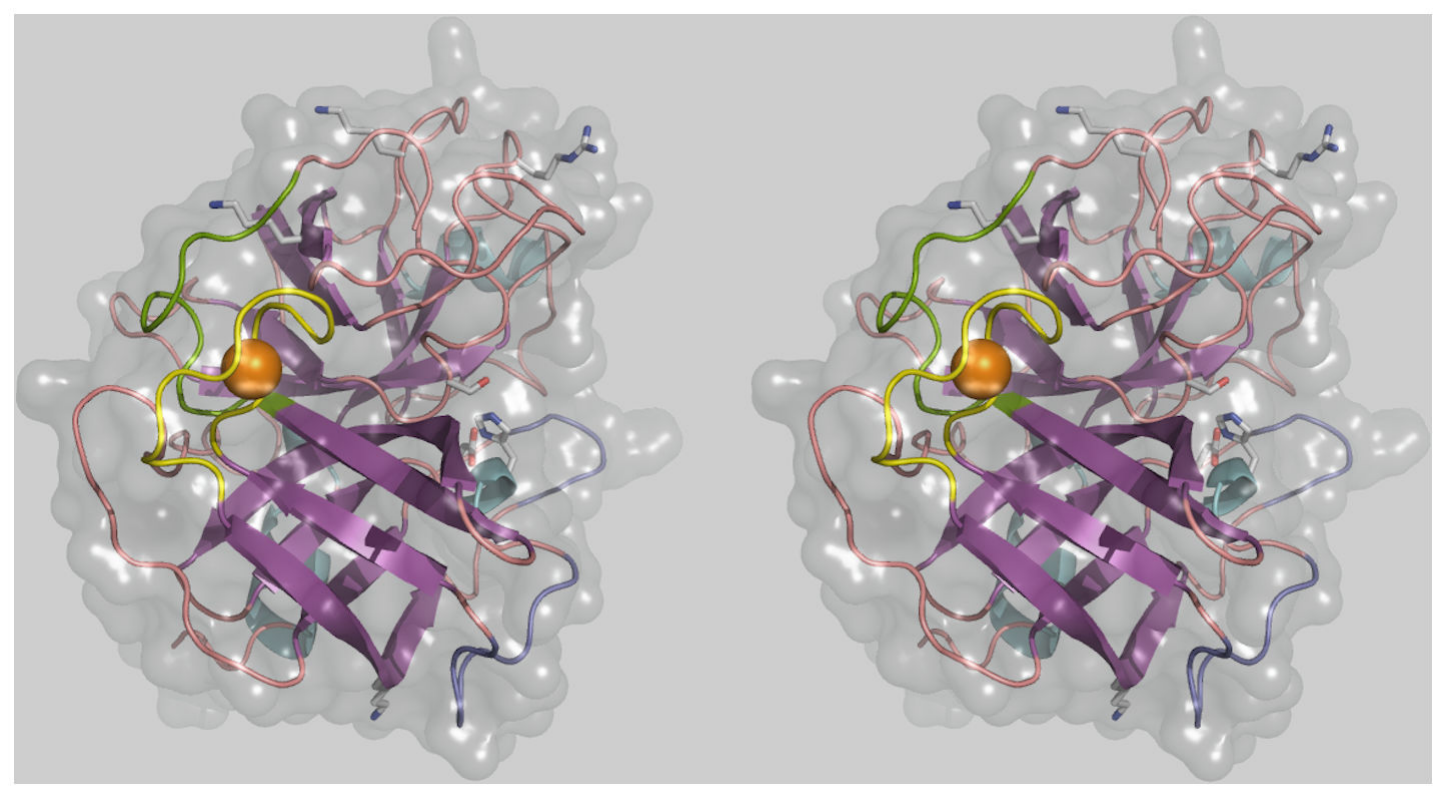
Wall-eye stereogram representation of the main features of the KT1 molecular model. The 3D molecular model of KT1 is represented in a wall-eye stereo view. β-sheets are represented in purple, α-helixes in cyan and loops in pink. The solvent accessible molecular surface is shown as a transparent gray envelope. Particular features of KT1 are highlighted. The TAL is showed in green and the CBL in yellow. The chelated Ca^2+^ atom is displayed as an orange sphere. Active site extending loops are shown in violet in the right-lower corner. The three active residues are represented at the middle-right of the figure. At the top, three of the putative cleavage sites amino acid residues are shown: K163, K197 and R230. The primary K96 cleavage site is shown at the bottom of the figure.

A structural model of KT1 was built (Figure 4) based on the most similar X-ray structure of the *Pontastacus* *leptodactylus* trypsin (CFT, PDB id. 2F91). *P* *. leptodactylus* lives between 9 and 18^°^C [54], and therefore CFT should have some cryophilic or intermediately cryophilic character.

KT1 and CFT have extended surface loops surrounding the active site, which are not observed in other trypsins. The function of these loops can be related to cold-adaptation by imparting more flexibility to the region surrounding the substrate-binding site. These loops also contain several solvent-exposed hydrophobic residues that shield the interaction of polar side chains with water molecules, imparting a better cold stability to the protein and favor substrate binding energetics at low temperatures [55,56].

KT1 and CFT have only 4 disulfide bridges instead of 6 in PT: C45-C61, C143-C210, C174-C189 and C200-C228 (amino acid residues are numbered according to the respective complete zymogen sequence order). Additionally, serine 54 in PT, located under the active site serine (PT-S195), is substituted by a cysteine residue (C57) in KT1 and CFT, with the bulkier sulfur atom of C57 in close contact with the C45-C61 disulfide bridge (see Text S1). These two features in KT1 and CFT could simultaneously provide flexibility and stability, another contributing factor to the cold-adaptation of these enzymes.

### Expression and production of active KT1

#### *E. coli* TB1*/*pMALc2E-KT1 expression system

**Figure 5:**
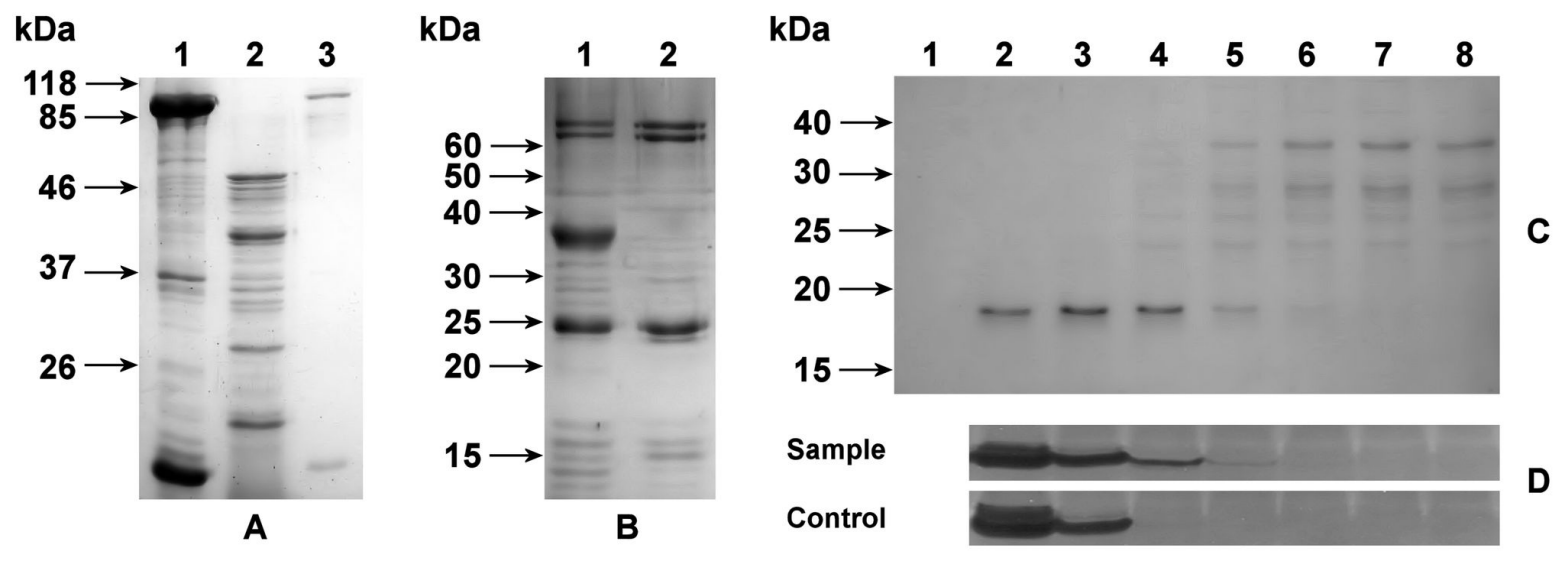
SDS-PAGE and zymographic analysis of the recombinant trypsinogen and its exogenous activation to produce active KT1. (A) SDS-PAGE of the products of the *E*. *coli* TB1/pMALc2E-KT1 expression system. Lane 1: purified inactive proteins from the soluble cytoplasmic cell fraction. Lane 2: purified proteins digested with enterokinase. Lane 3: purified expression control of uninduced cells. (B) SDS-PAGE of the products of the *E*. *coli* BL21(DE3)/pET22b-KT1 expression system. Lane 1: purified inactive proteins from the soluble cytoplasmic cell fraction. Lane 2: purified expression control of uninduced cells. (C) SDS-PAGE of digestions of the purified products of the *E*. *coli* BL21(DE3)/pET22b-KT1 expression system with different amounts of pig trypsin for the same incubation time. Lane 1: control with 5×10^-1^ U/ml of pig trypsin with no substrate. Lane 2: 5×10^-1^ U/ml. Lane 3: 1×10^-1^ U/ml. Lane 4: 2×10^-2^ U/ml. Lane 5: 4×10^-3^ U/ml. Lane 6: 8×10^-4^ U/ml. Lane 7: 1.6×10^-4^ U/ml. Lane 8: undigested control of the original sample with no pig trypsin. (D) Zymograms of the corresponding samples in (C). The control includes pig trypsin at the same amounts with no recombinant trypsinogen. Zymograms are presented as negative images for better clarity.

Recombinant MBP-KT1 fusion protein with a 6×His-tag was overproduced as a cytoplasmic soluble inactive polypeptide and was purified by a single IMAC step. The soluble MBP-KT1 accounted for ~40% of the total protein in the purified protein fraction (Figure 5A).

**Figure 6:**
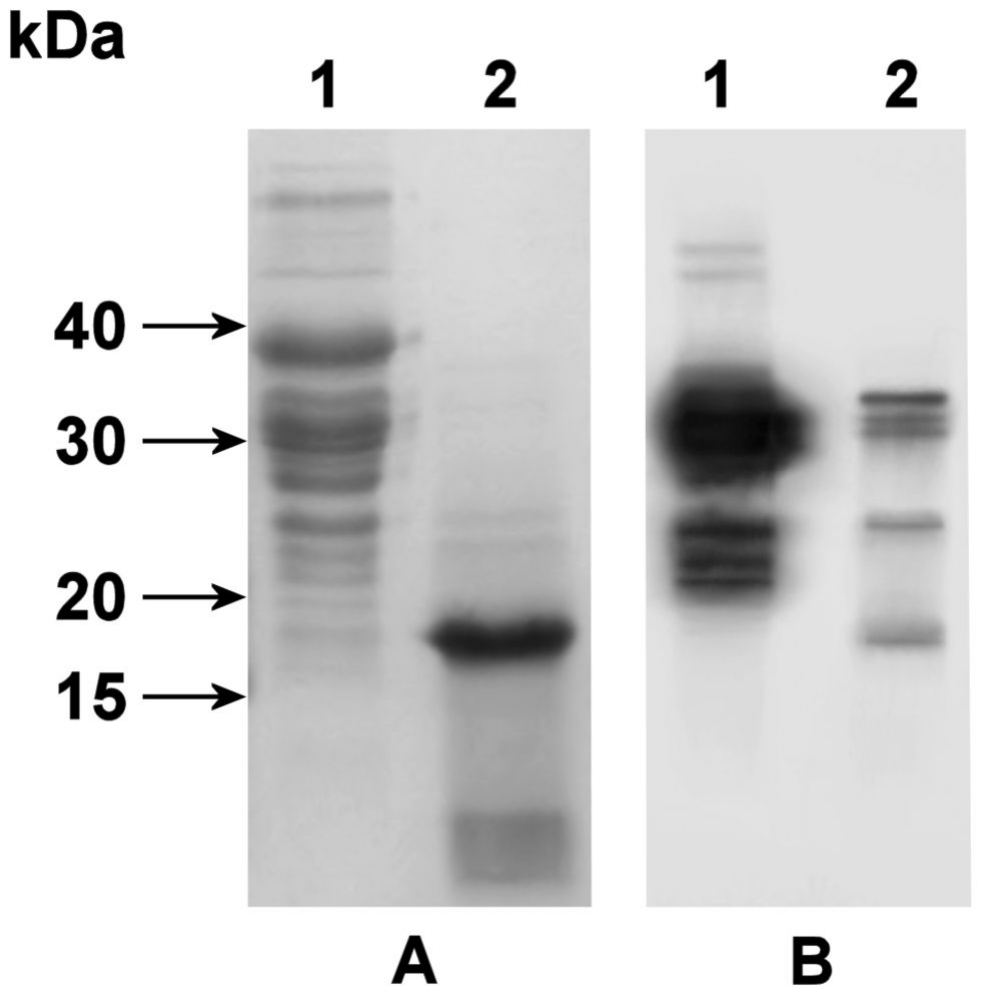
SDS-PAGE and Western blot analysis of the recombinant purified proteins and their post-activation products. (A) SDS-PAGE of the products of the *E*. *coli* BL21(DE3)/pET22b-KT1 expression system. Lane 1: purified inactive proteins from the soluble cytoplasmic cell fraction. Lane 2: end products after pig trypsin activation of the sample in lane 1. (B) Western blot of polyhistidine-tagged proteins present in the corresponding samples in (A).

The purified protein appears as a band of about 85 kDa in SDS-PAGE. Enterokinase digestion of MBP-KT1 yields two bands of around 47 and 38 kDa (Figure 5A). The first corresponds to the molecular mass of MBP and the second is the active KT1, which was confirmed by Western blot (Figure 6). KT1 presents an anomalous migration in SDS-PAGE, consistent with our previous results [30], which can be explained by its high content of acidic amino acids and very low pI [57–60]. Two other relevant bands around 26 and 17 kDa also appeared after enterokinase digestion (Figure 5A). These bands cannot be products of the highly specific enterokinase digestion, but are produced through proteolysis catalyzed by the activated trypsin.

#### *E. coli* BL21(DE3)/pET22b-KT1 expression system

KT1 was also expressed with an N-terminal periplasmic destination signal peptide and a C-terminal 6×His-tag. The recombinant protein was found mainly as insoluble inclusion bodies, with a minor cytoplasmic fraction, and an insignificant fraction in the periplasmic fraction of the cells (not shown). We were unable to recover active enzyme from inclusion bodies after solubilization and refolding. Therefore we used only enzyme from the purified cytoplasmic soluble fraction for the following experiments (Figure 5B). The recombinant IMAC-purified enzyme shows a major protein band around 38 kDa in SDS-PAGE.

### Demonstration of the autolytic susceptibility of KT1

KT1 purified from BL21(DE3)/pET22b was digested with various amounts of PT to release active KT1. One major band around 17 kDa and several bands around 26 kDa were detected after digestion (Figure 5C), coincident with bands that appeared after enterokinase digestion of MBP-KT1 (Figure 5A). No active KT1 band was clearly detected in SDS-PAGE, but short-lived protease activity was detected in zymograms after digestion (Figure 5D), unrelated to PT activity. Both pig trypsin and KT1 migrated as bands of less than about 20 kDa in non-denaturing PAGE zymograms.

A Western blot analysis was performed to identify bands containing the 6×His-tag of the recombinant protein after digestion. These results agree with the SDS-PAGE analysis, with a 38-kDa recombinant protein band and at least three minor bands between 20–26 kDa in the undigested sample (Figure 6). After digestion, two new polyhistidine-tagged bands appear around 26 kDa and 17-kDa. This demonstrates that the 17-kDa band observed in SDS-PAGE gels is a KT1 degradation product (Figure 6). The total intensity of the chemoluminescent signal decreases after digestion, which indicates that some of the polyhistidine tag is lost in low molecular weight species that migrate out of the gel.

### Ca^2+^ dependency of KT1

#### Experimental results

All known trypsins are dependent on Ca^2+^. This is due to the existence of a well-conserved flexible Ca^2+^-binding loop (CBL) close to the active site. The intrinsic mobility of this loop hinders the binding of the substrate. When Ca^2+^ is bound, the CBL stiffens and allows substrates to enter the active site.

**Figure 7:**
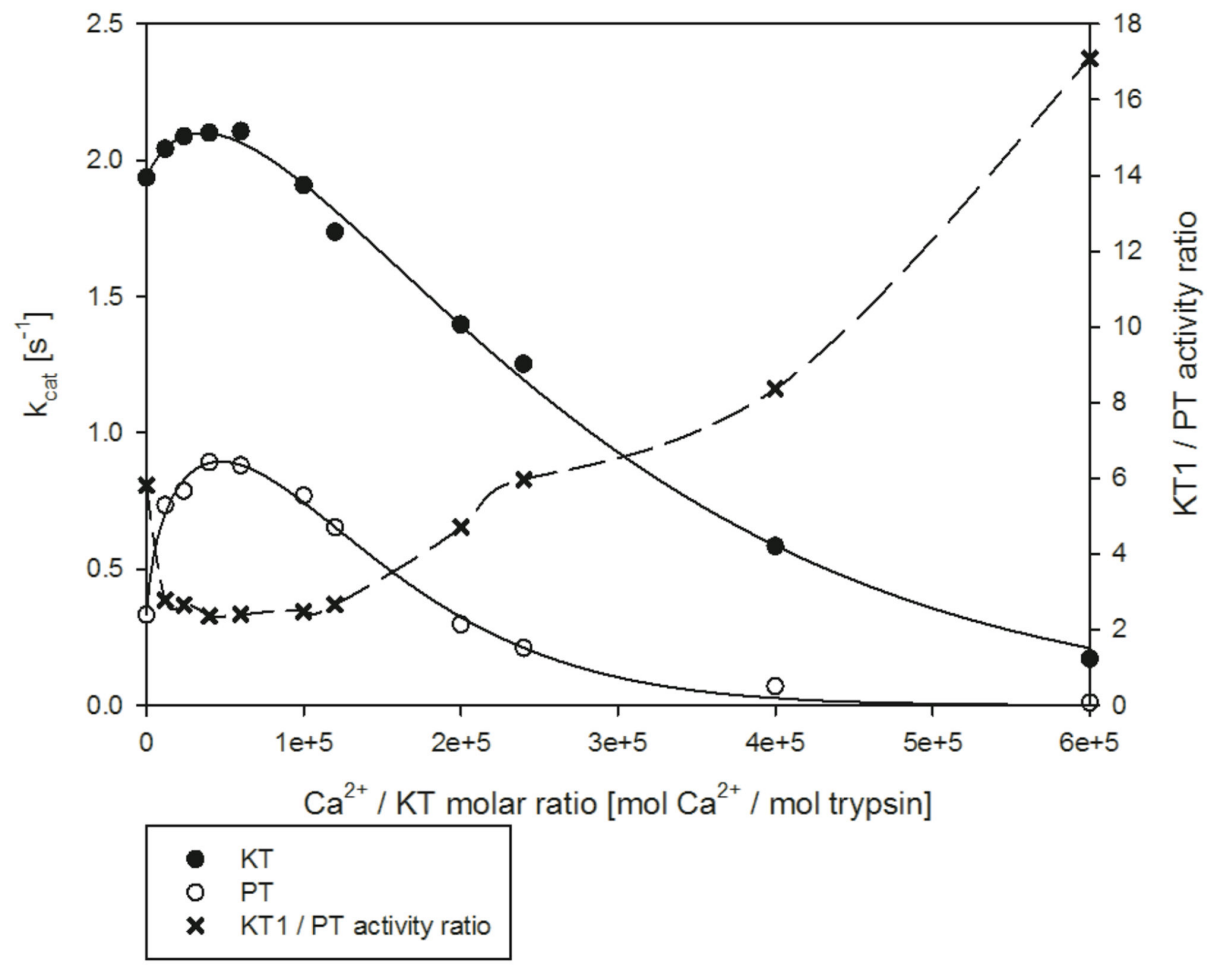
Trypsin-like activity versus Ca^2+^/enzyme molar ratio for KT1 and PT. A comparison plot between KT1 activity and PT activity at equivalent calcium/enzyme molar ratios (referred to on the right vertical axis) have been constructed to compare both calcium dependencies and extract more detailed information about the activity differences at low and high calcium concentrations.

Experiments were conducted to assess the Ca^2+^ dependency of KT1 activity compared with PT (Figure 7). As expected, KT1 has higher activities than PT at all Ca^2+^ concentrations. However, the KT1 activity profile presents a much higher activity in comparison with the maximum for lower Ca^2+^ concentrations and has a lower tendency to lose activity at higher Ca^2+^ concentrations. KT1 maximum activity is achieved at lower Ca^2+^/protein ratios, which points to a calcium-independent stabilization of the CBL. This indicates that KT1 has a lower dependency on Ca^2+^ for its activity.

#### Structural determinants of the unusual Ca^2+^ dependency in KT1

**Figure 8:**
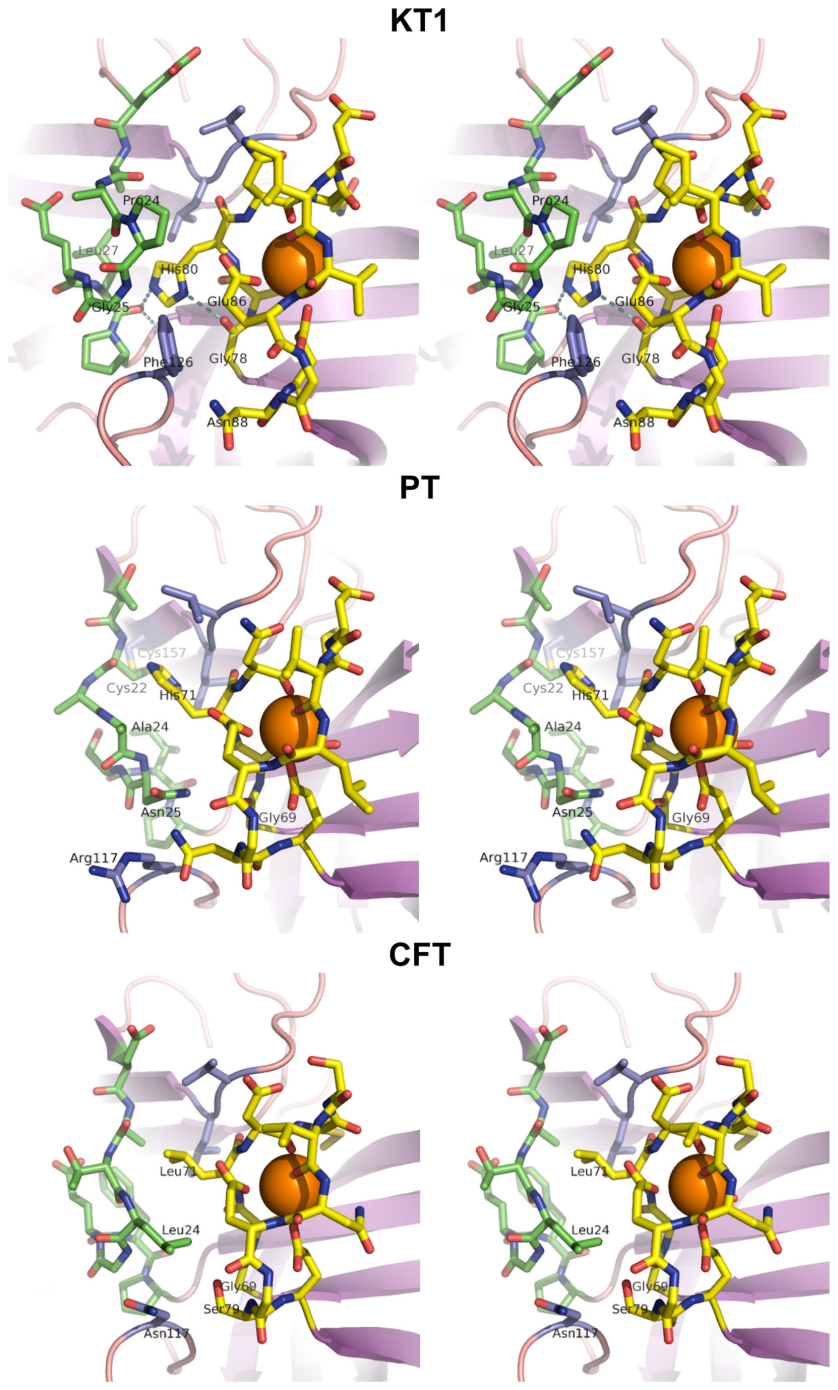
Wall-eye stereograms of the structural comparison between calcium binding loops of KT1, PT and CFT. Wall-stereo view of the molecular superposition of the calcium binding loops (yellow), trailing activation loops (green) and surrounding residues (violet) in the cryophilic krill trypsin 1 (KT1), the mesophilic pig trypsin (PT), and the homologous crayfish trypsin (CFT), showing the structural differences discussed in the text. CBLs and TALs are viewed from a point where the line of view is approximately perpendicular to the molecular surface of the protein. In this way, the closest residues to the viewer are the surface residues, and receding side chains are buried inside the protein structure. Amino acid side chains mentioned in the text are labeled. H80 hydrogen bonds in KT1 are shown as cyan dotted lines.

Differences between KT1 and PT CBLs (Figure 8) involve the replacement of PT solvent-exposed polar residues by aliphatic amino acids in KT1, which destabilize interactions with the solvent, but no mutations affect Ca^2+^-binding residues. However, at low temperatures where entropic contributions are more important than enthalpic interactions [55,56], these mutations could help to stabilize the loop faced with a highly structured solvent.

As a unique feature of KT1, mutations of the amino acids surrounding the CBL histidine residue (KT1-H80 ^≈^ PT-H71) accommodate the H80 side chain in a solvent excluding conformer. In PT, this histidine is solvent exposed and cannot rotate. The histidine side chain in KT1 can form two tight stabilizing hydrogen bonds with the carbonyl groups of L27 (belonging to the trailing activation loop, TAL) and G78 (in the CBL). In CFT, more closely related to KT1, H80 is replaced by a leucine (L71), which is unable to form hydrogen bonds with the solvent or with internal residues.

At low temperatures, the formation of buried intermolecular hydrogen bonds by H80 side chain in KT1, preventing water molecules to be trapped inside the protein and providing and enthalpic contribution to folding, could be more favorable than being exposed to the solvent, this assisted by the simultaneous exposure of hydrophobic surface in the native folded state [61–66]. CBL surface in KT1 includes the exposed large hydrophobic side chain of F126 instead of polar residues. PT has a much more polar molecular surface around the CBL and TAL than KT1, while CFT is an intermediate case (Figure 8). Hence, this can stabilize the CBL region and prevent enthalpically-driven cold unfolding, a key strategy for cold-adaptation [61–67] (see Text S1 for a detailed analysis).

Other mutations in and around the KT1 CBL also contribute to further stabilize this loop. Interactions between the CBL and the TAL residues are particularly relevant. The buried H-bonds of G78, unsatisfied without the presence of H80 in KT1, the absence of PT-N25, the presence of the bulky side chain of F126, the appearance of P24 in the TAL, and the elimination of the PT C22-C57 bridge structurally determine the buried position of the H80 side chain in KT1, with no rotation possibility (see Figure 8 and Text S1).

#### Energetic contribution of histidine and hydrophobic residues on the CBL stability

Hydrogen bonding of the H80 side chain in KT1 and the KT1-exclusive substitutions make possible the formation of a compact solvent-excluding core that stabilizes the CBL in the absence of Ca^2+^ and hence could be responsible for the decreased calcium dependency of KT1. This could be part of an evolutionary adaptation to low temperatures and higher salinity environments, since the substitutions in this protein section also make it less thermodynamically favorable to cold denaturation.

The added stability of these substitutions was assessed through a molecular dynamics analysis and equilibration energy calculations for the TAL and CBL with no Ca^2+^ binding in PT, CFT and KT1. Energy calculations demonstrated that without Ca^2+^ the CBL is more stable in KT1 than in PT or CFT (Table S2). The calculated total energies for CBLs and TALs in PT and CFT are ~50% and ~500% larger than those of KT1 in the absence of Ca^2+^. Since the energy calculation method does not consider explicit solvent molecules, entropic contributions at temperatures near the water freezing point cannot be correctly estimated [62,63,66], but these calculations are considered valid for temperatures near 20^°^ C.

### Autolysis stabilization

#### Structural determinants of KT1 autolytic susceptibility

The marked autolysis susceptibility of KT1 makes sense for a highly active trypsin to avoid self-digestion of the enzyme-producing tissues [68–71], but is not in agreement with reported cases of crustacean trypsins [72]. Ca^2+^ binding-induced stabilization has been demonstrated to provide protection against autolysis in mesophilic trypsins [68,69]. In KT1, this region is stable independently from Ca^2+^ binding. Additionally, KT1 lacks the equivalent to R117 in PT, one of the main autolysis sites in mammalian trypsins [73]. Hence, the autolysis mechanism of KT1 must be connected to other positively charged exposed residues on the protein surface.

Unlike pig trypsin, which has 14 basic residues, KT1 has only four auto-cleavable positively charged residues: K96, K163, K197 and R230 (roughly equivalent to K87, K159, K188 and K224 in PT).

In contrast to the other putative cleavage sites, K96 is not stabilized by salt bridges or nearby disulfide bonds, its side chain is exposed (30%), and is followed by an exposed peptide bond in a convex β-sheet bend (Figure 4). Considering the disulfide bond stabilization of the protein fold, only cleavage at K96 disrupts the catalytic triad. Therefore, K96 is structurally the most likely primary autolysis site in KT1, followed by R230 in order of importance (see Text S1 for a detailed structural analysis). These predictions are in agreement with the experimental results (Figures 5, 6). The only KT1 cleavage pattern able to produce the main ~17 kDa band observed after complete autolysis is consistent with cleavage at K96 (and possibly also R230). A single K96 cleavage can give a slightly more massive band at around 18.5 kDa, which would be difficult to distinguish from the 17-kDa band. This 18.5 kDa band still conserves the 6×His-tag of the recombinant construct, which is lost with cleavage at R230.

#### Construction of autolysis-stable mutant proteins

**Figure 9:**
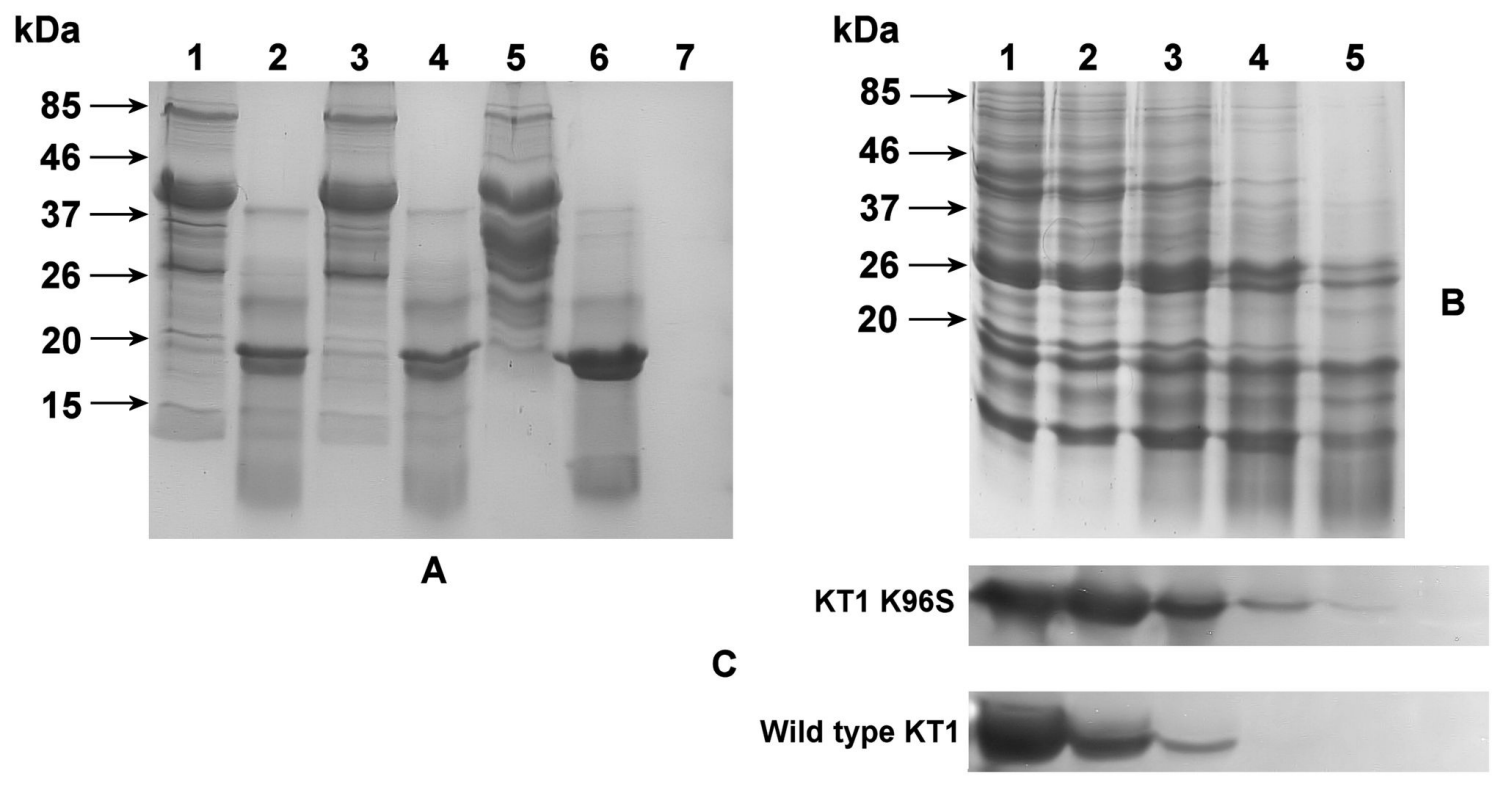
SDS-PAGE and zymographic analysis of KT1 mutants. (A) SDS-PAGE of the undigested and digested wild type KT1 and K96H and K96H+R230E mutants. Lane 1: purified K96H mutant. Lane 2: digested K96H mutant. Lane 3: purified K96H+R230E mutant. Lane 4: digested K96H+R230E mutant. Lane 5: purified wild type KT1. Lane 6: digested wild type KT1. Lane 7: PT used for digestion (1×10^-1^ U/ml). (B) SDS-PAGE of the digestion of the K96S mutant with different amounts of PT for digestion. Lane 1: no PT added. Lane 2: 8×10^-4^ U PT/ml. Lane 3: 4×10^-3^ U PT/ml. Lane 4: 2×10^-2^ U PT/ml. Lane 5: 1×10^-1^ U PT/ml. (C) Zymograms of the corresponding digestion of mutant samples in (B) compared to the digestion of wild type KT1 under the same conditions. Zymograms are presented as negative images for better clarity.

Mutants were constructed to remove the identified autolysis sites. MOSST [40] was used to predict non-disrupting amino acid substitutions, which were subsequently confirmed by SDM [41] to be stabilizing mutations. A single K96H mutation was introduced to remove the primary K96 cleavage site with no structural disruption, while a double K96H+R230E mutation was designed to eliminate the two most likely cleavage sites. Both variants showed a similar digestion pattern, as shown in Figure 9A, which demonstrates that the removal of the R230 cleavage site does not alter protein autolysis. Surprisingly, the elimination of the K96 positive formal charge does not impart autolytic stability to KT1.

Andante [39] and PROPKA3 [74] were used to predict the side chain conformation and pK_a_ of the residues at position 96. PROPKA3 results (pK_a_(K96) = 10.30; pK_a_(H96) = 5.83) do not absolutely preclude the presence of a positive charge on H96 in the experimental conditions. Hence, a third K96S neutral variant was constructed, using the second non-disruptive MOSST amino acid replacement suggestion for this position. This new variant showed the best autolysis resistance and was the only active variant detectable in zymograms (Figure 9B-C). A better digestion pattern was observed for this variant, with clearly observable bands in SDS-PAGE gels at around 26 kDa, which corresponds to the active enzyme species. The 17-kDa degradation band is less intense. Enzymatically active bands were also detected in zymograms (which were not observed in the K96H variants; data not shown). Through comparison of the zymogram band intensity (Figure 9C) at the same activating PT catalyst concentrations, we estimate that the mutant KT1-K96S is ~25 times more stable than the wild type enzyme under the same conditions.

## Conclusions

We purified and characterized a new krill trypsin with a remarkably high activity between 4 and 42^°^C and a broad range of activity versus pH, both desirable features for biotechnological applications. Thermodynamically and kinetically, the enzyme is unequivocally a cold-adapted or cryophilic trypsin.

We cloned two trypsin isoform gene sequences in krill, KT1 and KT4. Homology and phylogenetic analyses indicated that cold adaptation in this enzyme family is related to small changes in the entire protein. Extended surface loops surrounding the active site, less disulfide bridging and a serine to cysteine mutation under the active site in the protein core have been postulated as putative cryophilicity determinants. These are mechanisms that increase the flexibility of the protein and we have shown that they optimize the interaction with the solvent and the stability of the molecule at low temperatures.

After activation of the purified recombinant KT1 zymogen, several trypsin degradation products were detected and trypsin activity quickly decayed. The degradation pattern produced distinguishable end products. This natural susceptibility of KT1 to autolysis could be related to the presence of higher molecular flexibility or unstructured molecular regions associated with cold adaptation. Physiologically, this autolytic susceptibility can be interpreted as an evolutionary safekeeping mechanism to avoid self-digestion in the event of an undesired activation of a trypsin before secretion, a strategy that is transversal to all trypsin-producing organisms.

Although all trypsins are activated by Ca^2+^ through stabilization of a calcium-binding loop (CBT), we have shown that the psychrophilic KT1 shows an unusual Ca^2+^-activity dependency curve, in which Ca^2+^ concentration does not have a strong effect on trypsin activity. We demonstrated that this is due to the intrinsic structural stabilization of the CBT independent from Ca^2+^ chelation caused by specific amino acid residues present in KT1, namely H80, F126 and P24, and the absence of a C22-C57 disulfide bridge.

The intrinsically stable CBL also prevents enzyme inactivation by an autolytic cleavage at Lys197, a common autolytic control mechanism for mesophilic trypsins. We experimentally demonstrated that the inactivation mechanism for KT1 is mainly mediated by cleavage at K96. A K96H mutation did not greatly decrease this cleavage, but a neutral K96S mutation produced a 25-fold increase in autolytic stability. This allowed the observation of clear active bands in zymograms that were not detectable in similar conditions for the wild type enzyme.

Clearly, cold adaptation, Ca^2+^ dependence and autolytic stability in trypsins are related phenomena that are determined by the same structural features and hence they cannot be interpreted separately. These effects are related to particular point mutations or insertions in the protein sequence. Antarctic krill, and probably other low-temperature dwelling crustaceans, represent an evolutionarily divergent source of enzymes that have evolved these mutations to achieve high catalytic activity and structural stability at low temperatures, but simultaneously conserving tightly controlled inactivation mechanisms to avoid self-digestion. These adaptive molecular mechanisms seem to have evolved differently from other trypsins described so far.

Profiting from these evolutionary adaptations, we mutated the KT1 krill trypsin gene according to our design goals to obtain a novel proteolytic tool for biotechnological applications.

## Supporting Information

Text S1 **Supplementary material.**
(DOCX)

Table S1 **Thermodynamic parameters of enzyme activities.**
(DOCX)

Table S2 **Energy calculation results in kJ/mol for the trailing activation loop and the calcium binding loop for PT, CFT and KT1.**
The results are shown by amino acid residue contribution, as total energy for each loop.
(DOCX)
