# Supplementary material for: Cold Adaptation, Ca2+ Dependency and Autolytic Stability Are Related Features in a Highly Active Cold-Adapted Trypsin Resistant to Autoproteolysis Engineered for Biotechnological Applications
Source: PLoS One. 2013 Aug 12;8(8):e72355. doi: 10.1371/journal.pone.0072355 (PMC3741176; doi:10.1371/journal.pone.0072355)
Supplement: Text S1 — Supplementary material. (DOCX) [file pone.0072355.s001.docx]

**Cold adaptation, Ca^2+^ dependency and autolytic stability are related features in a highly active cold-adapted trypsin resistant to autoproteolysis engineered for biotechnological applications**

*Alvaro Olivera-Nappa^§^, Fernando Reyes, Barbara A. Andrews, Juan A. Asenjo*

Centre for Biochemical Engineering and Biotechnology, Department of Chemical Engineering and Biotechnology, University of Chile, Santiago, Chile.

# ^§^ E-mail: aolivera@ing.uchile.cl

# Supplementary material

## Structural model of the KT1 gene product

*Influence of the replacement of S54 in PT by C57 in KT1 and CFT*

Serine 54 in PT is buried under the active site serine (PT-S195). In KT1 and CFT, S54 is substituted by a cysteine residue (C57). This substitution has two direct effects: first, the bulkier sulfur atom of C57 instead of the oxygen atom of S54, locally dilates the hydrophobic core, and second, the stabilizing hydrogen bonding of the S54 side chain oxygen present in PT is lost in KT1. These two facts can be directly responsible for a higher mobility of the catalytic serine and histidine residues, which can be energetically favorable at low temperatures to overcome the energetic burden of the hydrolytic process. A higher mobility of these two residues can help to fine-tune the distances between them, the substrate and the catalytic aspartate, thus counteracting the lower molecular motions expected at low temperatures, in order to alter the nucleophilicity of the catalytic serine residue in each catalytic step.

The analysis of the molecular structure of KT1 shows also an unexpected possibility regarding the influence of the C57 mutation. In the molecular model, the sulfur atom of C57 is in close contact with the C45-C61 disulfide bridge (~3.3 Å). This close proximity, and the fact that the C57 side chain is completely buried inside the protein raises the possibility of the existence of an equilibrium-like redox hydrogen exchange between the sulfur of C57 and the sulfur atoms of this disulfide bond. Therefore, transient disulfide bridges could be formed between C45-C61, C45-C57 and C57-C61. Although a mechanism like this has not been previously described, we propose that it could provide a greater mobility to the catalytic histidine, at the same time providing flexibility and stability to KT1 and CFT active sites, which could be another contributing factor to the cold-adaptation of these enzymes. This remains to be further studied in the future.

## Ca^2+^ dependency of KT1

*Structural analysis of mutations around H80 in KT1*

CBLs superimpose very well in PT and KT1. However, the surrounding environment is very different. The most striking feature of KT1 CBL substitutions are mutations of the amino acids surrounding the CBL histidine residue (KT1-H80 ≈ PT-H71) that accommodate the voluminous polar histidine side chain in a solvent excluding conformer. In PT, this histidine is solvent exposed and cannot rotate. Due to mutations in KT1, the histidine side chain can putatively have two different conformations: one completely exposed to the solvent and another one forming two tight stabilizing hydrogen bonds with the carbonyl groups of L27 (trailing activation loop, TAL) and G78 (CBL). Interestingly, H80 is a leucine (L71) in the more closely related CFT that was used as a template to build the structural model of KT1. This leucine residue is unable to form hydrogen bonds with the solvent or with internal residues.

At cold temperatures, the establishment of buried intermolecular hydrogen bonds for H80 could be more favorable than the same hydrogen bond donors being exposed to the solvent, when hydrophobic surface is exposed in the native folded state at the same time. This structure stabilizing mechanism can be observed in KT1, where the hydrophobic side chain of F126 replaces a polar exposed surface around the CBL. This side chain is in the position occupied by solvent exposed polar R117 in PT and N117 and L24 in CFT. Interestingly, R117 is the main autolysis site in mammalian trypsins. PT has a much more polar molecular surface around the CBL and TAL than KT1, while CFT is intermediate (Fig.8). Hence, this can stabilize the CBL region and prevent enthalpically-driven cold unfolding, a key strategy for cold-adaptation.

The hydrogen bonding of H80 makes possible formation of a compact solvent-excluding core that rigidizes the CBL without the need of a Ca^2+^ ion to stabilize the structure by bridging different loops. Other mutations in and around the KT1 CBL can also contribute to further stabilize this loop. The interactions between the CBL and the TAL residues are very interesting. The TAL in KT1 has a different position relative to the CBL, intermediate between the position of this loop in PT and CFT. The stabilization of G78 in the CBL seems to be relevant. PT-G69 (≈ KT1-G78) in PT CBL is stabilized by hydrogen bonds to N25, but this asparagine is mutated to a glycine preceded by a proline residue in KT1 (P24-G25), which are sterically unable to interact with G78. In CFT, G69 is stabilized by S79 in the same CBL. The equivalent residue in KT1 is N88, but F126 in KT1 creates a hydrophobic exposed patch that hinders the rotation of N88 to interact with and stabilize G78. However, this structural arrangement keeps the Ca^2+^-chelating E86 in place, hindering its rotation. The appearance of P24 (L24 in CFT, A24 in PT) in the nearby TAL also contributes to restrict the movement of this region of the molecule in the absence of Ca^2+^. In addition, this side-chain replacement and the elimination in KT1 of the PT C22-C157 disulfide bridge structurally determine the buried position of the H80 side chain, with no rotation possibility. Taken together with the thermodynamic considerations previously exposed, this rules out the putative exposed H80 conformation, in agreement with model calculations.

## Autolysis stabilization

*Structural analysis of the possible autolysis sites in KT1 other than K96*

KT1 has only four auto-cleavable positively charged resides: K96, K163, K197 and R230 (roughly equivalent to K87, K159, K188 and K224 in PT). K188 in PT (KT1-K197) has been identified as one of the autolysis sites in the highly homologous human and rat trypsinogens-I. However, it was demonstrated that in the presence of 5 mM Ca^2+^, the cleavage at this site is negligible [80]. This is due to the stabilization of the Ca^2+^ binding loop. In the case of KT1, the CBT is stable without Ca^2+^, and the K197-D198 peptide bond is buried (in fact, D198 forms the oxyanion hole of the enzyme) and is protected from unfolding by two nearby disulfide bridges at C189 and C200. Therefore, it is unlikely that this site could be a target for trypsin autolysis.

Similarly, K163 and R230 in KT1 are most probably not primary autolysis sites. K163 is stabilized by a salt bridge with E26 in the TAL. The K163 side chain is buried deep inside the protein, *i.e.* 21% exposure vs. ~30% for the other basic residues in the protein. Additionally, the K163 carbonyl group is in an inaccessible concave surface zone in a β-sheet structure. Similarly, R230 is stabilized by a salt bridge with E153, has a P in the next position and is stabilized by the nearby disulfide bridge at C200-C228, which connects with the structurally stable oxyanion hole. However, the R230 carbonyl group is in a convex loop surface zone, and this could make it more susceptible to be attacked.

Taking into account the former analysis, the analysis of the K96 and the experimental results discussed in the main article, we ruled out K197 and K163 as possible autolysis sites and determined that K96 is the most likely primary autolysis site in KT1, followed by R230 in order of importance.
