# Supplementary material for: Cold Adaptation, Ca2+ Dependency and Autolytic Stability Are Related Features in a Highly Active Cold-Adapted Trypsin Resistant to Autoproteolysis Engineered for Biotechnological Applications
Source: PLoS One. 2013 Aug 12;8(8):e72355. doi: 10.1371/journal.pone.0072355 (PMC3741176; doi:10.1371/journal.pone.0072355)
Supplement: Table S1 — Thermodynamic parameters of enzyme activities. (DOCX) [file pone.0072355.s002.docx]

**Cold adaptation, Ca^2+^ dependency and autolytic stability are related features in a highly active cold-adapted trypsin resistant to autoproteolysis engineered for biotechnological applications**

*Alvaro Olivera-Nappa^§^, Fernando Reyes, Barbara A. Andrews, Juan A. Asenjo*

Centre for Biochemical Engineering and Biotechnology, Department of Chemical Engineering and Biotechnology, University of Chile, Santiago, Chile.

# ^§^ E-mail: aolivera@ing.uchile.cl

# Table S1

Thermodynamic parameters of enzyme activities

|  |  |  | |  | **Krill trypsin** | | | | |  | **Pig trypsin** | | | | |  | **Difference between the psychrophilic and mesophilic enzymes** | | | |  |
| --- | --- | --- | --- | --- | --- | --- | --- | --- | --- | --- | --- | --- | --- | --- | --- | --- | --- | --- | --- | --- | --- |
| **Enzyme** | |  | **Temperature** | |  | E_a_ | ΔG^#^ | ΔH^#^ | TΔS^#^ | |  | E_a_ | ΔG^#^ | ΔH^#^ | TΔS^#^ | |  | Δ(ΔG^#^)_p-m_ | Δ(ΔH^#^)_p-m_ | TΔ(ΔS^#^)_p-m_ | |
| **substrate** | |  | **(ºC)** | |  | (kJ/mol) | (kJ/mol) | (kJ/mol) | (kJ/mol) | |  | (kJ/mol) | (kJ/mol) | (kJ/mol) | (kJ/mol) | |  | (kJ/mol) | (kJ/mol) | (kJ/mol) | |
| **Casein** | |  | 20 | |  | 33.6 | 74.1 | 31.1 | -43.0 | |  | 47.1 | 75.5 | 44.6 | -30.8 | |  | -1.4 | -13.5 | -12.1 | |
| **BAPNA** | |  | 20 | |  | 27.7 | 70.1 | 25.2 | -44.9 | |  | 45.0 | 71.5 | 42.6 | -28.9 | |  | -1.3 | -17.3 | -16.0 | |
