# Supplementary material for: Cold Adaptation, Ca2+ Dependency and Autolytic Stability Are Related Features in a Highly Active Cold-Adapted Trypsin Resistant to Autoproteolysis Engineered for Biotechnological Applications
Source: PLoS One. 2013 Aug 12;8(8):e72355. doi: 10.1371/journal.pone.0072355 (PMC3741176; doi:10.1371/journal.pone.0072355)
Supplement: Table S2 — Energy calculation results in kJ/mol for the trailing activation loop and the calcium binding loop for PT, CFT and KT1. The results are shown by amino acid residue contribution, as total energy for each loop. (DOCX) [file pone.0072355.s003.docx]

**Cold adaptation, Ca^2+^ dependency and autolytic stability are related features in a highly active cold-adapted trypsin resistant to autoproteolysis engineered for biotechnological applications**

*Alvaro Olivera-Nappa^§^, Fernando Reyes, Barbara A. Andrews, Juan A. Asenjo*

Centre for Biochemical Engineering and Biotechnology, Department of Chemical Engineering and Biotechnology, University of Chile, Santiago, Chile.

# ^§^ E-mail: aolivera@ing.uchile.cl

# Table S2

Energy calculation results in kJ/mol for the trailing activation loop and the calcium binding loop for PT, CFT and KT1. The results are shown by amino acid residue contribution, as total energy for each loop.

| **Trailing activation loop** | | | | | |
| --- | --- | --- | --- | --- | --- |
| **PT** | | **CFT** | | **KT1** | |
| Residue | Energy | Residue | Energy | Residue | Energy |
| T | -28.163 | D | 0.547 | E | -16.353 |
| C | -15.894 | A | -19.459 | A | -27.783 |
| A | -2.433 | T | 5.818 | A | 2.044 |
| A | 0.532 | L | 32.301 | P | 89.413 |
| N | -165.054 | G | 41.31 | G | 35.196 |
| S | -16.968 | E | -17.273 | E | -16.225 |
| I | 38.022 | F | 16.084 | L | 6.464 |
| P | -9.347 | P | -8.463 | P | -29.329 |
| **TAL total** | **-199.305** |  | **50.865** |  | **43.427** |
|  |  |  |  |  |  |
| **Calcium binding loop** | | | | | |
| **PT** | | **CFT** | | **KT1** | |
| Residue | Energy | Residue | Energy | Residue | Energy |
| G | 18.073 | G | 17.301 | G | 10.583 |
| E | -27.157 | E | -25.586 | E | -6.199 |
| H | -5.616 | L | -40.62 | H | -37.752 |
| N | -202.641 | D | -19.146 | N | -196.149 |
| I | -25.054 | M | -18.457 | Q | -173.658 |
| D | -7.114 | S | -3.014 | D | -10.89 |
| V | -1.626 | V | 2.242 | I | -5.347 |
| L | -5.846 | N | -151.966 | V | -1.928 |
| E | 26.08 | E | 97.797 | E | 22.844 |
| G | 58.087 | G | 65.812 | G | 53.303 |
| N | -148.956 | S | -3.452 | N | -154.762 |
| E | -22.767 | E | -20.186 | E | -16.837 |
| **CBL total** | **-344.537** |  | **-99.275** |  | **-516.792** |
|  |  |  |  |  |  |
| **TAL+CBL total energy** | | | | | |
| **PT** | | **CFT** | | **KT1** | |
| -543.842 | | -48.41 | | -473.365 | |
